# Cell culture models for studying the development of Barrett’s esophagus: a systematic review

**DOI:** 10.1007/s13402-012-0076-6

**Published:** 2012-04-03

**Authors:** P. Bus, P. D. Siersema, J. W. P. M. van Baal

**Affiliations:** Department of Gastroenterology and Hepatology, University Medical Center Utrecht, Heidelberglaan 100, 3584 CX Utrecht, The Netherlands

**Keywords:** Bile salts, Acid, Reflux, Barrett’s esophagus, *In vitro* model, Cell lines

## Abstract

**Background:**

Barrett’s esophagus (BE) is a premalignant condition caused by chronic gastroesophageal reflux. BE patients have an increased risk of developing esophageal adenocarcinoma (EAC). As many aspects of this condition are still unknown, there is a need for *in vitro* models to study BE development.

**Aim:**

To review the literature on cell lines and incubation conditions for studying BE development.

**Methods:**

A literature search was performed using PubMed, EMBASE and the Cochrane library, combining the words esophagus, cell line, culture, Barrett’s, bile, acid, exposure, reflux and adenocarcinoma.

**Results:**

A wide range of cell lines and incubation conditions to study BE development have been reported. The most commonly used cell lines are derived from epithelium from patients with BE or EAC. A 25-minute incubation with 200 μM bile salts induced cell proliferation and Akt phosphorylation. However, increased CDX2 and MUC2 expression was only observed with longer incubations or higher bile salt concentrations. Two-hundred μM bile at pH 6 showed a higher toxicity to EAC cells than the same concentration at pH 7. Multiple 5-minute exposures with 200 μM bile at pH 4 or pH 7 increased CK8/18 and COX2 in BE epithelial cells.

**Conclusions:**

Two-hundred μM conjugated primary or secondary bile salts at pH 4 for multiple short exposures is able to induce BE specific factors in BE cell lines. In SQ and EAC cell lines; however, higher concentrations of secondary bile salts for 8 h are needed to induce BE specific molecules. Due to the high variability in reported methods, it is difficult to determine the most effective *in vitro* setup for studying the development of BE.

## Introduction

Physiological reflux of gastric content from the stomach into the esophagus occurs in the majority of individuals [1]. When these reflux episodes occur more frequently, it will lead to gastroesophageal reflux disease (GERD). In patients with GERD, the refluxed fluid contains predominantly acid, which is exposed to the esophageal mucosa for a longer period of time compared to the physiologic situation [2]. GERD patients have also been shown to have higher concentrations of bile salts in the refluxate compared to healthy volunteers [3]. According to several studies, patients with GERD have an increased risk of developing Barrett’s esophagus (BE) [4–6]. In BE, the normal squamous epithelium of the distal esophagus is replaced by intestinal metaplastic columnar-lined epithelium, containing goblet cells [7]. BE is a pre-malignant condition that predisposes to esophageal adenocarcinoma (EAC), with an incidence of approximately 0.5% per patient year [8–10]. This incidence is rising faster than that of any other malignancy in the Western world [11]. The prognosis of patients with EAC is mostly infaust with a 5-year survival rate of 10–15% [12].

It has been shown that the refluxate of BE patients also consists of higher concentrations of acid and bile salts compared to patients with GERD [3, 13]. Since long, it has been suggested that acid and bile salts play a predominant role in inducing BE and, when this condition is present, it may stimulate progression towards EAC. The exact mechanism remains however unknown.

Barrett’s epithelium is characterized by the expression of several factors that distinguishes it from the normally present squamous epithelium in the distal esophagus, for example Caudal type Homeobox transcription factor 2 (CDX2), Cyclin D1, Cyclooxygenase-2 (COX2), Mucin 2 (MUC2) and Bone morphogenetic protein 4 (BMP4) [14–19]. Other factors that are expressed in BE include Nuclear Factor-κB (NF-κB), Mucin 1 (MUC1), c-myc and the inflammatory cytokines Interleukin-8 (IL-8) and IL-1β [20–24]. Moreover, the phosphorylation of ERK1/2 is decreased, which is known to regulate proliferation and apoptosis [25]. Of special interest are the cytokeratins (CKs), which are often used to differentiate between normal squamous esophageal epithelium and BE [18]. CK10 and 13 are expressed in normal squamous esophageal epithelium, while CK7, 8, 18 and 20 expression is typically found in BE [16, 18].

The metaplastic process causing esophageal squamous epithelial cells to transform into intestinal metaplastic columnar-lined epithelium (BE), followed by progression to EAC is complex [26]. In order to understand the pathogenetic mechanisms that induce BE, it is important to know how bile salts and/or acid induce these changes and whether these processes can be inhibited. Since there is no good animal model available, an *in vitro* model, in which reflux of acid, bile or a combination, and its effects can be simulated and inhibited, would be useful. Over the last few years, several studies using various types of cell cultures to investigate the underlying mechanisms of bile and/or acid involved in BE development have been published. In this review, we will discuss these cell culture models with specific emphasis on cell lines, reflux components and incubation conditions.

## Methods

A systematic search of the English-language literature indexed in PubMed, the Cochrane library and EMBASE was conducted that included a combination of the following search queries: esophagus, Barrett’s, cell line, culture, bile, acid, exposure, reflux and adenocarcinoma. The search was performed for the period 1990 until October 2011. In addition, a manual search of citations in relevant articles was performed. In order to limit the focus of this review, papers using *ex vivo* cultures, colon cancer cell lines and esophageal squamous carcinoma cell lines, papers reporting patient studies, animal studies and previous reviews were excluded. Our query resulted in 159 papers. Fourty-six publications finally met the inclusion criteria.

## Results

### Cell lines

Cell lines have the advantage that large numbers can be grown and are expected to be a more or less stable model. In addition, cell lines can be cultured for a longer period than *ex vivo* cultures of esophageal squamous epithelial or Barrett’s epithelial cells. In a study by Palanca-Wessels *et al.*, the majority of primary (*ex vivo*) cultures of Barrett’s epithelium were maintained for 1 week, whereas only a small number of cultures extended for longer periods, up to 4 months [27].

A disadvantage of cell lines is however that they can differentiate towards another cell type or that cross-contamination may occur, particularly when several cell lines are cultured simultaneously at the same site. Several studies using cells that had differentiated towards another cell type have been published [28, 29]. Another disadvantage is that cultured cell lines do not interact with other cell types or stroma, as these are not present in the culture, but likely play a role in overall functioning in the *in vivo* situation.

In the literature, various cell lines have been reported for studying the development of BE, ranging from normal esophageal epithelial cell lines to BE and EAC cells. It is uncertain whether the cell lines that have been used in the literature indeed contain the specific cell characteristics as suggested. For example, Feagins *et al.* reported that their non-neoplastic Barrett’s epithelial (BAR) cells behaved in fact as normal fibroblast cells and doubted their Barrett’s origin [30]. Furthermore, Avissar *et al.* suggested that the EAC cell line Seg-1 had characteristics that are typical of lung epithelium [31]. Moreover, Alvarez *et al.* questioned the origin of several esophageal adenocarcinoma cell lines, among which were Seg-1 cells [32]. These cell lines have been widely used to study BE development [2, 17, 33–40].

From literature, it is known that the transformation of Barrett’s metaplasia towards dysplastic epithelium and EAC is accompanied by an increase in genomic alterations and instability [41, 42]. This suggests that BE cell lines contain fewer genomic alterations; which can be of potential influence in an *in vitro* model, as the EAC cell lines will respond differently to bile and / or acid exposure than the BE cell lines. This might be an important factor to keep in mind, while setting up cell culture experiments. It is difficult to determine which cell line is the ideal *in vitro* model for induction of factors normally expressed in BE. This will often depend on the aim of the study and should be repeatedly determined for each experiment. Based on the *in vitro* results in the literature using the Seg-1 cell line incubated with bile salts at low or neutral pH, this cell line could be an appropriate cell line to use; however, it has been suggested that these cells are not from an esophageal origin, but rather from lung epithelium [31]. Therefore, we suggest using a squamous esophageal epithelial (Het-1A), Barrett’s epithelial (e.g. BAR-T) or EAC cell line (e.g. OE33) (Table 1).

**Table 1:** Cell lines for studying pathogenetic mechanisms of Barrett’s esophagus

Cell lines	Cell type
Het-1A	Squamous esophageal epithelial cell line
NES-B3T, NES-B10T	Cell line derived from squamous tissue from GERD patient with BE
NES-G2T, NES-G4T	Cell line derived from squamous tissue from GERD patient without BE
CP-D, BAR-T	Barrett’s epithelial cells
OE33, OE19, FLO-1, SKGT4	Esophageal adenocarcinoma cell line

Various cell lines used to study BE in an *in vitro culture* model along with the type of cell

### Bile salts at neutral pH

The type and concentration of bile salts in the gastro-esophageal refluxate of BE patients has been determined in several studies [13, 43]. Nehra *et al.* reported that the median bile salt concentration in BE patients was 181 μM compared to 124 μM in patients with erosive esophagitis and 14 μM in patients with minimal mucosal injury [13]. The percentage of secondary bile salts was also higher in the refluxate of BE patients, compared to GERD patients and controls [13]. Secondary bile salts (e.g. deoxycholic acid (DCA), lithocholic acid (LCA) and their conjugated forms) are bile salts that are dehydroxylated and unionized at neutral pH and therefore more lipophilic, which may cause cell membrane rupture. Primary bile salts are cholic acid (CA) and chenodeoxycholic acid (CDCA) and these are the products of cholesterol metabolism. Takahashi *et al.* found that the bile hydrophobicity ratio is a predictor of developing BE, in which the ratio is calculated by dividing the concentration of hydrophobic bile acids (LCA, glycolithocholic acid (GLCA) and taurolithocholic acid (TLCA)) through the concentration of hydrophilic bile acids (ursodeoxycholic acid (UDCA), glycol-ursodeoxycholic acid (GUDCA) and tauro-ursodeoxycholic acid (TUDCA)); LCA, GLCA and TLCA are in higher concentrations found in the BE reflux than in the reflux of non-BE patients [43]. Nehra *et al.* also found that in BE patients, higher concentrations of conjugated bile acids are present [13]. Conjugation of bile acids leads to different toxic properties, with unconjugated bile acids and glycine conjugated bile salts having a higher toxicity than taurine conjugated bile acids. Bile acid toxicity has been investigated in Het-1A cells by Sharma *et al.* who found that CDCA, DCA and LCA dramatically decreased cell viability, while the conjugated forms of DCA and CDCA showed no change in viability [44]. Moreover, from LCA and DCA it is known that they play a role in colorectal carcinogenesis [45]. Based on these data, we can conclude that the most important bile acids in the development of BE are LCA, DCA, CDCA and glycine conjugated bile acids.

In several studies, reflux was simulated by exposing cell lines to different types of bile salts. The most frequently used bile salts are the secondary bile salt DCA (1–1,000 μM) and the conjugated primary bile salt glycochenodeoxycholic acid (GCDCA) (50–1,000 μM) [17, 36, 37, 46–48]. Bile salt mixtures have been used in concentrations ranging from 100–940 μM, and mostly consist of conjugated primary bile salts [34, 38, 48–51].

An increase in NF-κB activity was found in OE33 cells upon incubation with a secondary bile salt for 2 h, while an even higher increase was measured after 8 h of incubation [47]. Incubation of BAR-T, Het-1A and OE33 cells with the same secondary bile salt resulted in increased production of reactive oxygen species (ROS) and nitric oxide (NO), DNA damage and NF-κB activity [52–54].

Increased IL-6 secretion was found upon incubation of OE33 cells with a primary bile salt [55]. Increased MUC2 and CDX2 expression was found upon exposure to 300 and 1,000 μM DCA in Seg-1 cells [17], and lower concentrations of this bile salt (50 and 100 μM) were found to increase MUC2 and NF-κB expression [56]. An increase in MUC2 and CDX2 expression has been measured in Het-1A cells after exposure to 1,000 μM DCA [17]. It has also been reported that lower concentrations (500 μM) of (un-)conjugated primary bile salts were already sufficient to increase CDX2 expression in Het-1A cells [57]. CDX2 expression was also increased in OE19 cells (an EAC cell line) upon incubation with 100 μM DCA; however, CDX2 protein levels were not changed [58].

Seg-1 cells showed a significant increase in proliferation rate, measured by cell count, upon incubation with a conjugated primary bile acid, with the highest increase in cell numbers at a concentration of 500 μM [37]. Akt plays a role in the inhibition of apoptosis and the promotion of proliferation; its activity was found to be 3-fold increased in Seg-1 cells after exposure to the same conjugated primary bile acid, and after exposure to 300 μM DCA [36, 59].

Keeping the normal clinical situation in mind, it seems most relevant to study the effect of bile salt mixtures in an *in vitro* model. We found 6 studies that used bile salt mixtures to simulate the gastroesophageal refluxate, an overview of these studies, together with the toxicity profile is shown in Table 2 [34, 38, 44, 48–51]. One bile mixture consisted for 80% of conjugated bile acids, incubation with this mixture did not show oxidative stress in EAC, BE and SQ cell lines [34]. The bile mixtures in the other 5 studies consisted of only conjugated bile acids. Incubation of Seg-1 cells with these mixtures caused an increase in CDX2 mRNA, but did not affect cell viability and oxidative stress [34, 38, 49]. Incubation of Het-1A cells with these mixtures decreased cell viability and increased CDX2 and MUC1 mRNA [48, 49, 51]. While FLO-1 cells (an EAC cell line) showed a decrease in cell viability upon incubation with these mixtures [48].

**Table 2:** Bile mixtures to study pathogenetic mechanisms of Barrett’s esophagus

Article	Bile mix	Cell line	Incubation period	Findings	Conjugation status	Toxicity profile [44]
Dvorak *et al.*[34]	20 μM GCA + 20 μM TCA + 20 μM GDCA + 20 μM GCDCA + 20 μM DCA	Seg-1, Barrett’s cells and Het-1A	10 min	No increase in oxidative stress	80%	GCA, TCA, GDCA, GCDCA: +; DCA: -
Hao *et al.* [38]	0.54 mM NGCA + 0.1 mM NTCA + 0.2 mM GCA + 0.1 mM NTCDCA	Seg-1	1 h	Cell number ↑; No change in cell viability	100%	All: +
Jolly *et al.* [48]	NGCA + GCA + NTCA + NTCDCA (250 μM and higher)	Het-1A	3 h	Cell viability↓	100%	All: +
	NGCA + GCA + NTCA + NTCDCA (500 μM and higher)	FLO-1	3 h	Cell viability↓	100%	All: +
Liu *et al.* [49]	GCA + TCA + GCDCA + TCDCA + GDCA + TDCA (20:3:15:3:6:1) (400 μM)	Het-1A and Seg-1	3 * 10 min/day for 7 days	Increase in CDX2 mRNA	100%	All: +
Huo *et al.* [50]	GCA, TCA, GCDCA, TCDCA, GDCA, TDCA (400 μM) (20:3:15:3:6:1) at pH 4 or 7.2	NES-G2T, NES-G4T; NES-B3T, NES-B10T	3 * 10 min/day for 7 days	Increase in CDX2 mRNA and protein in NES-B3T and NES-B10T at pH 4 and pH 7.2	100%	All: +
Van Roon *et al.* [51]	GCA + TCA + GCDCA + TCDCA + GDCA + TDCA (20:3:15:3:6:1) (120 μM)	Het-1A	3 * 5 min/day for 3 days	Increase in MUC1 mRNA	100%	All: +

Various articles investigated the pathogenetic mechanisms of Barrett’s esophagus, along with the bile mixtures, cell lines, incubation periods and the observed effect on cell viability, cell growth, oxidative stress and CDX2 expression

*GCA* glycocholic acid, *TCA* taurocholic acid, *GDCA* glycodeoxycholic acid, *GCDCA* glycochenodeoxycholic acid, *DCA* deoxycholic acid, *NGCA* sodium glycocholic acid, *NTCA* sodium taurocholic acid, *NTCDCA* sodium taurochenodeoxycholic acid. NES-G2T and NES-G4T, squamous esophageal epithelium cell line from GERD patients without BE; NES-B3T and NES-B10T, esophageal epithelium cell line from GERD patients with BE. CDX2, caudal type homeobox transcription factor 2

Toxicity profile: +, no effect or increased cell viability in bile salt toxicity test of Het-1A cells; -, decrease in cell viability below 50%

Exposure of Seg-1 cells, BAR cells and Het-1A cells to 200 μM conjugated primary bile salts increased cell proliferation and Akt phosphorylation [36, 37]. NF-κB activation, MUC2 and CDX2 expression were however only induced after exposing cells to higher concentrations of secondary bile salts, i.e. 300 and 1,000 μM [17, 47]. Taken together, these results suggest that low concentrations of conjugated primary bile salts or higher concentrations of secondary bile salts can be used for incubation to see a BE specific effect. The bile mixtures that have been reported in the literature are mainly conjugated primary bile acids, with the results showing that these induce BE specific effects as well [34, 38, 48, 49, 51].

### Acid

A refluxate with a pH of 2 is rather common in BE patients [2]. However, the pH that is in contact with the esophageal epithelium is unlikely to be that low, as epithelial cells *in vivo* have a mucus layer at the apical side, which is able to neutralize at least partly the acid environment. When *in vitro* cell lines are used, this protection is not present, which makes it likely that a pH of 2, when used in cell culture experiments, is more harmful than it actually is *in vivo*.

In the reported studies, cells were exposed to pHs ranging from pH 2 to pH 7, with proliferation and oxidative stress measured as endpoints [2, 30, 34, 38, 39, 47, 48, 60]. In Het-1A and Seg-1 cells, exposure to pH 2 or 4 for 1 min caused an increase in ROS formation, while another study showed that a 10-min exposure to pH 4 did not result in increased oxidative stress [2, 34]. In addition, incubation of Seg-1 cells at pH 3.5 for 20 min did not result in increased cell proliferation or COX2 expression [38]. In contrast, Morgan *et al.* showed that the same incubation schedule caused suppression of the apoptosis-related protease, Caspase-9, and upregulation of Proliferating Cell Nuclear Antigen [39]. Moreover, a 10-minute exposure to pH 4 caused ROS production and DNA damage in BAR-T cells [61]. Total cell numbers were found to be decreased, when BAR cells were incubated twice with medium acidified to pH 4 for 3 min, at intervals of 10, 60 or 120 min [30]. An increase in DNA damage was found in Het-1A cells after a 30-minutes exposure to pH 4.5 [48]. NF-κB activity was increased in OE33 cells upon incubation with pH 4 for 1 h [47]. Moreover, a 1-hour exposure to pH 6 caused an increase in Early Growth Response 1 gene (EGR1) expression in these cells. This gene encodes for a transcription factor which regulates cell proliferation and apoptosis [60].

According to the studies summarized above, both pH 2 and pH 3.5 damage cells and therefore should not be used in an *in vitro* model. In contrast, incubation conditions at pH 4 and pH 6 results in measurable effects. This was further illustrated by the finding that 1-hour exposure to pH 4 in OE33 cells increased NF-κB activity, which has also been reported in *ex vivo* cultures of Barrett’s tissue and EAC tissue, and in *in vivo* biopsies [47].

It can be concluded that incubating cell lines with pH 4 seems to a large extent comparable to what is found *in vivo*; therefore this pH should be used in *in vitro* models when bile salts are not included in the incubation schedule.

### Bile salts at acidic pH

According to Nehra *et al.* reflux in BE patients mainly consists of bile salts at low or neutral pH [13]. Therefore, the combination of bile salts and acid is widely used in *in vitro* models. Bile salts can either be ionized or unionized depending on the pH of the solution and the pKa value of the specific bile salt [62]. The pKa value of unconjugated bile salts is between 5.2–6.2, while glycine conjugates have a pKa of 3.8–4.8 and taurine conjugates a pKa of around 2 [63, 64]. Lowering the pH towards these values may unionize bile salts, which makes them less soluble and able to enter the epithelial cells and influence intracellular pathways. Conjugation of bile acids results in a higher amount of bile salts in its soluble and ionized form at any given pH, so a higher concentration of bile acids can reflux into the esophagus [65]. The pH range in which most bile acids exist in their soluble, unionized form is pH 3–6. At these pHs, bile acids can enter epithelial cells and affect important pathways [65]. At lower pH, bile acids are precipitated and no longer damaging the epithelium, while at higher pH, bile acids exist in their non-damaging ionized form [66]. This suggests that unconjugated and glycine conjugated bile acids are most toxic between pH 3-6, while for taurine conjugated bile acids the pH needs to be approximately 2 to be able to pass the cell membrane.

Nine studies have reported on the combination of bile salts and acid to mimic reflux [34, 46, 48–50;67–69] (Table 3). Jolly *et al.* reported that a pH 4.5 alone increased DNA damage in Het-1A cells, which did not further increase upon exposure to pH 4.5 in combination with a bile salt mixture [48]. Similar results were found by Jenkins *et al.*, who found that incubation of OE33 cells with 200 μM DCA at pH 5.5 showed the same increase in DNA damage as DCA at pH 7 [46]. These results suggest that low pH or bile salts alone are as toxic to these cells as bile salts at low pH. However, incubation of the same cells with 100–400 μM DCA at pH 6 enhanced toxicity to cells compared to DCA at pH 7 [46]. An increase in DNA damage was also reported in Het-1A cells upon exposure to a 100 μM bile mixture at pH 4, with no difference if treated with acid alone [34]. In CP-A, CP-D (two Barrett’s cell lines, metaplastic and dysplastic, respectively) and Seg-1 cells, DNA damage was however only seen after exposure to the combination of 100 μM bile mixture at pH 4 [34, 69]. Feagins *et al.* showed that ROS production is increased in BAR-T, NES-B3T and in NES-G2T cell lines (the latter two are cell lines derived from squamous tissue from GERD patients with or without BE, respectively) upon incubation with a conjugated bile salt at pH4; these cell lines used however different mechanisms to increase this production [67]. These data suggest that already significant changes occur in the transition of reflux esophagitis towards BE.

**Table 3:** Bile salts at acidic pH to study pathogenetic mechanisms of Barrett’s esophagus

Article	Cell line	Bile	pH	Incubation period	Findings	Conjugation status	Toxicity profile [44]
Feagins *et al.* [67]	BAR-T cells	200 μM GCDCA	3, 4, 5	3 min	ROS levels increased	100%	+
	NES-B3T NES-G2T	200 μM GCDCA	4	3 min	ROS levels increased	100%	+
Dvorak *et al.* [34]	Seg-1 Het-1A	100 μM bile mix (GCA + TCA + GDCA + GCDCA + DCA)	4	10 min	Oxidative DNA damage	80%	GCA, TCA, GDCA, GCDCA: +; DCA: -
Jolly *et al.*[48]	Het-1A FLO-1	1, 10, 100 μM bile mix (NGCA + GCA + NTCA + NTCDCA)	4.5	15 min	DNA damage	100%	All: +
Jenkins *et al.* [46]	OE33	100 μM – 200 μM DCA	5.5	1 h	Increase in micronuclei	0%	-
	OE33	100 μM – 400 μM DCA	6	1 h	Increase in micronuclei	0%	-
Liu *et al.* [49]	Het-1A SEG-1	400 μM bile mix (GCA + TCA + GCDCA + TCDCA + GDCA + TDCA (20:3:15:3:6:1))	4	3 * 10 min/day for 7 days	Increase in CDX2 mRNA	100%	All: +
Huo *et al.* [50]	NES-G2T, NES-G4T; NES-B3T, NES-B10T	GCA, TCA, GCDCA, TCDCA, GDCA, TDCA (400 μM) (20:3:15:3:6:1)	4	3 * 10 min/day for 7 days	Increase in CDX2 mRNA and protein in NES-B3T and NES-B10T	100%	All: +
Goldman *et al.* [68]	Het-1A	200 μM bile mix (GCA + TCA + GDCA + GCDCA + DCA)	5.5	Initially 10 min/ once or twice a week; built up till 120 min.	Increase in villin, CDX2, CK8/18, COX2 expression and IL-6 secretion	80%	GCA, TCA, GDCA, GCDCA: +; DCA: -

An overview of the articles studying the effect of acid and bile on the expression pattern and behaviour of esophageal cell lines

*NES-B3T* esophageal epithelium cell line from GERD patients with BE, *NES-G2T* squamous esophageal epithelium cell line from GERD patients without BE, *GCDCA* glycochenodeoxycholic acid, *ROS* reactive oxygen species, *GCA* glycocholic acid, *TCA* taurocholic acid, *GDCA* glycodeoxycholic acid, *DCA* deoxycholic acid, *NGCA* sodium glycocholic acid, *NTCA* sodium taurocholic acid, *NTCDCA* sodium taurochenodeoxycholic acid, *CDX2* caudal type homeobox transcription factor 2. *CK* cytokeratin, *COX2* cyclo-oxygenase-2, *IL-6* Interleukin-6, Toxicity profile: +, no effect or increased cell viability in bile salt toxicity test of Het-1A cells; -, decrease in cell viability below 50%

The most frequently reported bile salt concentrations *in vitro* are 100 μM and 200 μM conjugated primary and secondary bile salts, and 100 and 200 μM bile mixture at pH 4–6, consisting for 80% out of conjugated bile salts and for 60% out of primary bile salts. An increased toxicity has been found in OE33 cells upon a 1-hour incubation with 100 or 200 μM secondary bile salt at pH 5.5 and in Het-1A cells upon incubation with a 100 μM bile mixture at pH 4.5 for 15 min [46, 48].

It can be concluded from these results that the combination of 100 or 200 μM bile salts, either conjugated primary or unconjugated secondary bile salts, at pH 4 seems the most optimal and physiological culture condition for cell lines, as demonstrated by the observations that these conditions show a higher toxicity compared to bile salts at pH 7 [34, 46, 48].

### Incubation periods

It is not sure whether BE develops after prolonged and/or chronic exposure of esophageal squamous epithelial cells to a refluxate containing bile salts and acid, or, alternatively, whether just one period of severe reflux causing severe damage to the esophageal lining is causing this premalignant condition [70]. Based on 24-hour pH monitoring, the incubation period that is most comparable to the reflux episodes *in vivo* is approximately 3 min [71]. However, in order to obtain comparable results as in the *in vivo* situation, incubation periods should probably be longer; Since, even negative controls experience reflux episodes, measured by pH monitoring [13, 72].

In the literature, various different incubation periods have been reported, ranging from 1 min to up to 48 h [2, 17, 24, 34, 36–39, 46–48, 55, 57, 60, 67, 73–77]. The shorter incubation periods, ranging from 1–20 min, are summarized in Table 4. A 3-minute exposure to 200 μM conjugated primary bile salt caused an increase in oxidative stress in BAR-T, NES-G2T and NES-B3T cells, while an exposure of 10 min to a 100 μM bile mixture at pH 4 caused oxidative stress in Seg-1 cells [34, 67]. Exposure of OE33 cells to a secondary bile salt at pH 5 caused an increase in CDX2 expression [75]. An exposure of 15 min to pH 4.5 alone caused a higher increase in DNA damage in FLO-1 cells and Het-1A cells than exposure to a 100 μM bile mixture at pH 4.5 using the same incubation time [48].

**Table 4:** The effect of the different durations of bile salt and/or acid incubations on the behaviour of the cell lines

Time (min)	Squamous esophageal epithelial cells (Het-1A)	Barrett’s epithelial cells (BAR-T)	Adenocarcinoma cells (Seg-1, FLO-1, OE33, OE21)
1	pH 2, pH 4 → oxidative stress [2]		pH 2 → oxidative stress [2]
3		200 μM GCA at pH 4 → oxidative stress [67]	
5		50 μM CDCA → cell proliferation ↑ [37]	50 – 100 μM TCA at pH 3-4.5 → ERK and p38 activation [74]
10			100 μM bile mix at pH 4 → oxidative stress [34]
			100 μM DCA at pH 5 → CDX2, VEGF ↑ [75]
15	100 μM bile mix at pH 4.5 →		100 μM bile mix at pH 4.5 → DNA damage [48]
	DNA damage [48]		pH 3.5 → no change in cell proliferation [38]
20			pH 3.5 → no change in cell proliferation [38]
			50–1000 μM GCDCA → cell proliferation↑ [36]

An overview of the changes measured upon one short exposure to bile salts and/or acid

*GCA* glycocholic acid, *CDCA* chenodeoxycholic acid, *TCA* taurocholic acid, *DCA* deoxycholic acid, *CDX2* caudal type homeobox transcription factor 2, *VEGF* vascular endothelial growth factor, *GCDCA* glycochenodeoxycholic acid

The results from longer incubation periods, from 1–48 h, are shown in Table 5.

**Table 5:** The effect of the different durations of bile salt and/or acid incubations on the behaviour of the cell lines

Time (hrs)	Squamous esophageal epithelial cells (Het-1A)	Adenocarcinoma cells (Seg-1, FLO-1, OE33, SKGT4)
1		940 μM bile mix → cell proliferation ↑ [38]
		100 or 200 μM DCA → DNA damage ↑ [46]
		pH 4 → NF-κB activity ↑ [47]
		pH 6-7 → EGR1 mRNA ↑ [60]
2		300 μM DCA → NF-κB activity ↑ [47]
3	250 μM bile mix → cell viability ↓ [48]	500 μM bile mix → cell viability ↓ [48]
4		1000 μM DCA → CDX2, MUC2 ↑ [17]
		300 μM DCA → COX2 ↑ [73]
		300 μM DCA → IL-8, IκB ↑ [77]
8	1000 μM DCA → MUC2, CDX2 ↑ [17]	300 μM DCA → MUC2, CDX2 ↑ [17]
18		50, 100 or 300 μM DCA, CDCA or TCA → NF-κB and MUC2 ↑ [56]
24	200, 500 μM CA, 500 μM GCA or 1000 μM DCA → CDX2 ↑ [57]	1000 μM DCA → CDX2, MUC2 ↑ [17]
		50, 100, 200 μM DCA → oxidative stress and micronuclei ↑ [46]
		500 μM TCA, 1000 μM TDCA, 500 μM TCDCA, 500 μM GCA or 50 μM DCA → MUC1 ↑ [76]
		100 μM DCA or CDCA with/without pH 4 → c-myc ↑ [24]
		100 μM DCA → CDX2, VEGF ↑ [75]
48		100 μM CDCA → IL-6 secretion ↑ [55]

An overview of the changes measured upon one longer exposure to bile salts and/or acid

*DCA* deoxycholic acid, *NF-κB* Nuclear Factor- κB, *EGR1* early growth response gene 1, *CDX2* caudal type homeobox transcription factor 2, *MUC2* mucin 2, *GCA* glycocholic acid, *CA* cholic acid. *COX2* cyclooxygensase-2, *IL-8* Interleukin-8, *CDCA* chenodeoxycholic acid, *TCA* taurocholic acid, *TDCA* taurodeoxycholic acid, *TCDCA* taurochenodeoxycholic acid, *GCA* glycocholic acid, *MUC1* mucin 1, *VEGF* vascular endothelial growth factor

Exposure times of 1 and 24 h have most frequently been used. The effects that are measured upon a 1-hour exposure to bile salts or acid are consistent with those occurring *in vivo*, such as an increase in NF-κB activity [47], cell proliferation [38] and EGR1 expression [60].

Reflux episodes occur more frequently in patients with GERD and BE compared to healthy controls [78], this suggests that multiple or pulsatile exposures of cell cultures to acid and/or bile salt seem to correspond most optimal to the clinical situation. This approach was used in five studies [30, 49, 51, 79, 80]. In one study, BAR-T cells were incubated with conjugated primary bile salts at pH 4, pH 6 and pH 7.4 for 5 min [79]. A single exposure did not result in a measurable effect, while daily exposures of 5 min for a period of 2–6 weeks made these cells showing a more colonic phenotype, characterized by expression of CK8/18, as well as a colonic epithelial protein, which is specifically expressed in colonic epithelial and BE cells [79]. Another study showed that daily 5-minute exposures of BAR-T cells to a conjugated primary bile salt for 22 weeks, increased the COX2 expression 10-fold [80]. The other studies showed that exposure of BAR cells to two 3-minute incubations of pH 4 at 10-, 60- or 120-minute intervals decreased cell numbers; that three 10-minute exposures per day to 400 μM bile mixture at acidic and neutral pH resulted in an increase in CDX2 expression in Het-1A and SEG-1 cells and that 5-minute exposures 3 times per day for 3 days to 120 μM bile mixture caused an increase in MUC1 expression in Het-1A cells [30, 49, 51].

In summary, repeated 3- or 5-minutes exposures or one single exposure of 1 h to bile salts and / or low pH result in BE specific changes *in* vitro.

## Summary

In Table 6, an overview of the *in vitro* changes in the several cell lines upon incubation with bile salts at low or neutral pH are shown, together with the changes known to occur in BE patients. In SQ cell lines, CDX2 and MUC2 mRNA is increased upon incubation with a secondary bile salt [17]. In Barrett’s cell lines either one exposure or multiple exposures to a conjugated primary bile salt, results in an upregulation of BE specific factors, e.g. COX2 [37, 79]. In an EAC cell line, one incubation with a secondary bile salt results in the upregulation of CDX2, MUC2 and NF-κB [17, 47]. In an *in vivo* model of BE in rats, in which BE is induced by performing an esophagogastroduodenal anastomosis, it has been shown that COX2, CDX2 and MUC2 is upregulated [81, 82]. Moreover, in BE biopsies it has been shown that CDX2, CK7/8/18/20, COX2 and MUC2 expression is increased, along with an increased NF-κB activation and an increased proliferation [15–18]. These changes also occur in the *in vitro* models mentioned in Table 6. Taken together, the *in vitro* models shown in Table 6 are the most ideal models that can be used to study the development of BE.

**Table 6:** *In vivo* and *in vitro* reflux episodes and their induced changes

	Barrett’s epithelium of BE patients	Squamous esophageal epithelial cell lines	Barrett’s cell lines		Esophageal adenocarcinoma cell lines
Exposure:	181 μM bile mix[13]	1000 μM DCA	200 μM GCDCA	200 μM GCDCA at pH4 or 7.4	300 μM DCA
Incubation period:	Multiple reflux episodes with an average duration of 2.6 min [71]	8 h	5 min	Multiple incubations of 5 min	8 h
Upregulation of:	CDX2	CDX2 [17]	Cell proliferation ↑ [37]	CK8, 18 [79]	CDX2 [17]
	CK7, 20, 8, 18	MUC2 [17]		COX2 [79]	MUC2 [17]
	COX2				NF-κB [47]
	MUC2				
	NF-κB				
	Cell proliferation ↑				

The effect of bile and different incubation periods on the expression pattern and behaviour of esophageal cell lines and the changes on the expression *in vivo*

*DCA* deoxycholic acid, *GCDCA* glycochenodeoxycholic acid, *CDX2* caudal type homeobox transcription factor 2, *CK* cytokeratin, *COX2* cyclo-oxygenase 2, *MUC2* mucin 2, *NF-κB* Nuclear Factor- κB

## Discussion

The last decades, both BE and EAC have dramatically risen in incidence [83]. Patients with BE have an increased risk of developing EAC, with an annual incidence of approximately 0.5% [8–11]. For this reason, endoscopic surveillance programs for BE patients have been developed to detect early stage EAC aiming to improve the prognosis of BE patients diagnosed with EAC [84].

It is currently largely unknown by which mechanism(s) BE develops. It has been suggested that at least partly a genetic basis is involved [85, 86]; however, the type and severity of gastroesophageal reflux is most probably involved as well [3, 13]. Unfortunately, an animal model that is clearly representing the situation in humans is not available. The most used animal models are variants of surgical rat models, which are physiologically not similar to humans and require long time periods to develop BE and EAC [87, 88]. Rodents also have keratinized epithelium and no submucosal glands, which is the opposite to the human epithelium [89]. In addition, there is a high mortality rate after the surgical procedure, a low incidence of BE lesions, uncertainty about the true nature of the lesion and the progression to malignancy is not similar to human malignant progression [90–92]. Finally, knocking out certain genes in rats is rather complicated, if not impossible.

Therefore, until there are animal models available that are validated for studying BE and EAC, we need to rely on *ex vivo* and *in vitro* models. The latter are easiest to use, as *in vitro* models have the advantage that they are widely available, can be cultured for longer periods and are not dependent on the presence of patients with BE. It is important that there is consensus on which *in vitro* model to use, in order to combine the results of different studies.

In this review, we reported a large variety of *in vitro* models that have been reported to induce factors normally expressed in BE. This review shows that many different cell lines and incubation conditions are available, and, in addition, the measured outcome parameters are often different. It is therefore difficult to conclude that there is a single most perfect *in vitro* model to study BE development.

It seems likely that the choice for a specific cell line to investigate pathogenetic mechanisms in BE largely depends on the research question. If the aim is to study the process of metaplasia of normal esophageal squamous epithelial cell to a Barrett’s like cell type, esophageal squamous epithelium cell lines, such as Het-1A cells, are most appropriate to use in an *in vitro* model. For other research questions, particularly the development of esophageal adenocarcinoma, BAR-T or OE33 cells can be used. It is, however, important to keep in mind that cell lines are usually transformed in order to keep the cells continuously growing, and therefore may behave differently compared to esophageal cells growing *in vivo*. Moreover, the microenvironment is also not present in an *in vitro* system, i.e., cells do not interact with other cell types, particularly inflammatory cells, or with products secreted by the surrounding stroma, i.e., growth factors and inflammatory mediators, which also play a role in the grow pattern and functioning of esophageal lining cells.

From the reviewed studies it can be concluded that *in vitro* bile salts at either a low or neutral pH are required to induce the changes that are seen in BE, such as NF-κB activation and increased cell proliferation. Based on our review, we suggest that a combination of bile salts and acid, i.e., 100 or 200 μM conjugated primary bile salts at pH 4 for 1 h, seems the most optimal culture condition to induce a Barrett’s like phenotype. This concentration of bile salts at low pH is similar to what is found in the refluxate of BE patients and has been shown to induce DNA damage [13, 34]. In addition, incubation with pH 4 alone increased NF-κB activity and incubation with 100 or 200 μM bile salt at neutral pH increased MUC2 and CDX2 expression [47, 56, 58]. An alternative experimental *in vitro* model includes multiple short exposures to bile salts and/or acid, which is comparable to the gastroesophageal reflux episodes that occur in GERD [49, 50, 79]. Moreover, multiple incubations with 200 μM bile salt at pH 4 induced the upregulation of CK8, CK18 and COX2 [79].

In conclusion, this data suggests that some selected *in vitro* cell line systems are able to induce the expression of markers that are specific for Barrett’s epithelium. When these models are used it may well be that our knowledge on the pathogenesis of the development of BE and its progression towards EAC can be improved.

## Abbreviations

GERD: Gastro-esophageal reflux disease
BE: Barrett’s esophagus
EAC: esophageal adenocarcinoma
CDX2: caudal type homeobox transcription factor 2
COX2: cyclooxygenase-2
MUC2: mucin 2
NF-κB: Nuclear Factor-κB
IL-8: Interleukin-8
MUC1: mucin 1
CK: cytokeratin
DCA: deoxycholic acid
ESCC: esophageal squamous cell cancer
LCA: lithocholic acid
GCDCA: glycochenodeoxycholic acid
CA: cholic acid
GCA: glycochenodeoxycholic acid
ROS: reactive oxygen species
CDCA: chenodeoxycholic acid
TCDCA: taurochenodeoxycholic acid
TCA: taurocholic acid
GDCA: glycodeoxycholic acid
TDCA: taurodeoxycholic acid
MCM4: mini-chromosome maintenance protein 4
EGR1: early growth response gene 1
VEGF: vascular endothelial cell growth factor
GLCA: glycolithocholic acid
TLCA: taurolithocholic acid
UDCA: ursodeoxycholic acid
GUDCA: glycol-ursodeoxycholic acid
TUDCA: tauro-ursodeoxycholic acid
NO: nitric oxide
